# Disproportionate vs. Proportionate Secondary Mitral Regurgitation: A Long-Term Pilot Analysis After Mitral Valve Surgery

**DOI:** 10.3390/jcm14103470

**Published:** 2025-05-15

**Authors:** Giovanni Alfonso Chiariello, Michele Di Mauro, Emmanuel Villa, Piergiorgio Bruno, Andrea Mazza, Natalia Pavone, Marialisa Nesta, Alberta Marcolini, Rudy Panzera, Andrea Armonia, Gaia De Angelis, Serena D’Avino, Antonio Nenna, Annalisa Pasquini, Massimo Massetti

**Affiliations:** 1 Department of Cardiovascular Sciences, Agostino Gemelli Foundation Polyclinic IRCCS, 00136 Rome, Italy; piergiorgio.bruno@policlinicogemelli.it (P.B.); andrea.mazza@policlinicogemelli.it (A.M.); natalia.pavone@policlinicogemelli.it (N.P.); marialisa.nesta@policlinicogemelli.it (M.N.); alberta.marcolini@guest.policlinicogemelli.it (A.M.); rudy.panzera01@icatt.it (R.P.); andrea.armonia@outlook.com (A.A.); gaia.deangelis03@icatt.it (G.D.A.); serena.davino@guest.policlinicogemelli.it (S.D.); annalisa.pasquini@policlinicogemelli.it (A.P.); massimo.massetti@policlinicogemelli.it (M.M.); 2 Faculty of Medicine and Surgery, Catholic University of the Sacred Heart, 00168 Rome, Italy; 3 Cardiovascular Research Institute, CARIM, 6229 ET Maastricht, The Netherlands; michele.dimauro@grupposynergo.com; 4 Casa di Cura Pierangeli, 65124 Pescara, Italy; 5 Department of Cardiovascular Surgery, Poliambulanza Foundation Hospital, 25124 Brescia, Italy; emmanuel.villa@poliambulanza.it; 6 Cardiac Surgery Unit, “AOU Maggiore della Carità” Hospital, 28100 Novara, Italy; antonio.nenna@maggioreosp.novara.it

**Keywords:** functional mitral regurgitation, mitral valve surgery, disproportionate mitral regurgitation, proportionate mitral regurgitation

## Abstract

**Objectives:** The treatment of secondary mitral regurgitation (MR) is still controversial. In 2019, a new conceptual framework was introduced, distinguishing between patients with a degree of MR “proportionate” to the left ventricular (LV) dilatation and patients in whom the severity of MR is “disproportionate” to the LV dilatation. The aim of this study was to compare the long-term outcome of patients with disproportionate vs. proportionate secondary MR who underwent mitral valve (MV) surgery. **Methods:** From January 2012 to June 2022, 96 patients with a preoperative diagnosis of pure secondary MR and LV dysfunction underwent MV surgery. The patients were divided in two groups, disproportionate vs. proportionate MR, according to echocardiographic parameters. A 5.2 (3.5–7.5) years complete clinical and echocardiographic follow-up was performed. **Results:** In the study period, 61 patients with disproportionate and 35 patients with proportionate MR underwent surgical MV repair or MV replacement. The thirty-day outcome was comparable in the two groups. At long-term follow-up, mortality was 5% in the disproportionate group vs. 11% in the proportionate group (*p* = 0.2), and cardiovascular mortality was 3% vs. 9%, respectively (0.5). Rehospitalization for heart failure was 6% vs. 20% (*p* = 0.04), and the rate of patients with New York Heart Association (NYHA) functional class ≥ III was 8% vs. 26%, respectively (*p* = 0.01). LV volumes were significantly higher in the proportionate group, thus presenting a lower LV ejection fraction (*p* < 0.001 and *p* = 0.03, respectively). No cases of recurrent MR have been observed. **Conclusions:** In this first exploratory analysis, patients with disproportionate secondary MR seem to present a possible benefit in terms of mortality and cardiovascular mortality, although not ones reaching statistical significance. Nevertheless, significant advantages were observed in terms of rehospitalization for heart failure, clinical status and symptoms, LV volumes, and LV function. Among patients referred to cardiac surgery, identifying the subset of patients with functional MR, who may obtain more significant advantages from surgery, seems relevant for patient selection, risk stratification, and to predict long-term outcomes.

## 1. Introduction

Secondary or functional mitral regurgitation (MR) refers to an altered systolic tethering of structurally normal mitral valve (MV) leaflets and the subvalvular apparatus, caused by global or localized abnormalities of left ventricular (LV) shape and motion [1,2,3]. The treatment of functional MR, largely related to LV dilatation and systolic dysfunction, is still controversial regarding the possible benefits of MV surgery. Identifying the subset of patients with functional MR who may benefit most from MV treatment seems crucial [1,2,3,4,5,6,7,8,9]. To evaluate the real severity of functional MR, the effective regurgitant orifice area (EROA) alone can be confounding. Indeed, it is necessary to consider the EROA in relation to the LV end-diastolic volume (LVEDV) [10]. Because of the conflicting results of the two trials on MitraClip (Abbott, Illinois, USA) in patients with functional MR in the MITRA-FR and COAPT trials [11,12], in 2019 a new conceptual framework was introduced by Grayburn PA et al. [13], with the attempt to reconcile the results of both trials. By considering the EROA/LVEDV ratio, it should be possible to distinguish patients with a degree of MR “proportionate” to the LV dilatation and patients in whom the severity of MR is “disproportionate” to the LV dilatation [13,14]. The aim of this study was to apply this recent framework to patients who had previously undergone MV surgery, by comparing the 30-day and long-term outcome, distinguishing between patients operated on for proportionate or disproportionate functional MR [13,14].

## 2. Materials and Methods

This study was an ambispective (retrospective–prospective), observational, single-center cohort study. Data from patients who underwent MV surgery were collected retrospectively, but a long-term clinical and echocardiographic follow-up was performed.

Comparison in terms of 30-day and long-term outcome between patients who underwent MV surgery from January 2012 to June 2022 for disproportionate vs. proportionate secondary MR was performed. The patients included in the study presented symptoms and signs of heart failure despite guidelines-directed medical therapy (GDMT) and cardiac resynchronization therapy (CRT) when indicated, showing a grade 2–3+ or 4+ functional MR (EROA > 20 mm^2^; regurgitant volume, RVol > 30 mL) and LV dysfunction, with left ventricular ejection fraction (LVEF) ≤ 55% and > 20% [13,15,16,17]. Patients with moderate MR underwent stress echocardiography. If the severity of MR increased and/or PASP (pulmonary artery systolic pressure) values increased (> 60 mmHg), patients were candidates for surgical treatment and included in the study. Patients presented chronic MR of ventricular origin with LV dysfunction; therefore, patients with functional MR of primary atrial origin, as patients with chronic atrial fibrillation, were excluded from this study. Patients referred for percutaneous treatment of MR, reinterventions, and patients who also underwent aortic valve surgery were excluded. Finally, only patients with pure secondary MR were considered. For this reason, patients with any sign of primary organic MR, detected by echocardiogram or at surgical inspection (degenerative, rheumatic, infective endocarditis, and others), or a mixed etiology (both organic and functional) were also excluded from this study.

A transthoracic echocardiogram was performed at admission, in the early postoperative course, before discharge, and at follow-up by a core lab of two experienced cardiologists dedicated to the echocardiographic evaluation of cardiac surgery patients. A transesophageal echocardiogram was performed if needed. The EROA was calculated using the PISA (proximal isovelocity surface area) method. The LVEDV and LVESV were calculated using the biplane Simpson’s method. Accordingly, the EF was calculated using the ratio [(LVEDV − LVESV)/LVEDV] × 100. The Rvol was calculated using the volumetric method.

Patients were divided into two groups, considering the ratio between the EROA and LVEDV. Patients with an EROA/LVEDV > to 0.15 mm^2^/mL were considered as being affected by “disproportionate MR”; in other words this group was composed of patients with a degree of MR excessively severe relative to the degree of LV dilatation. Patients with an EROA/LVEDV ≤ 0.15 mm^2^/mL were considered as being affected by proportionate MR, with a degree of MR proportionate to the concomitant LV dilatation [14]. Surgical indication was validated by the Institutional Heart Team and according to the European Society of Cardiology (ESC) Guidelines [18,19].

Surgical MV repair or replacement were performed by median sternotomy (12% of patients underwent a minimally invasive sternotomy with central arterial cannulation and central or peripheral bicaval cannulation for venous drainage), cardiopulmonary bypass (CPB), and crystalloid or blood–crystalloid cardioplegia. In all patients, the MV was exposed by a right atrium and trans-septal incision extended to the roof of the left atrium according to the Guiraudon approach [20]. Patients underwent MV repair by annular reshaping with a rigid or semi-rigid undersized prosthetic ring. MV replacement was performed, preserving the native MV leaflets and the subvalvular apparatus in all patients, and the prosthesis was implanted in an intra-annular position using multiple non-absorbable braided polyester sutures reinforced with pledgets, according to the anatomical features of the MV, the characteristics of the patient, and surgeons’ preferences. In patients who underwent MV repair or bioprosthesis implantation, postoperatively, subcutaneous enoxaparin was administered early after intervention, and subsequently Warfarin was added to the therapy. Once the international normalized ratio (INR) had reached 2.0–2.5, Warfarin was administered for three months. Subsequently, the patients required life-long acetyl-salicylic acid at 100 mg/day. Patients who underwent mechanical MV replacement received life-long Warfarin therapy.

The baseline preoperative data included age, sex, body mass index (BMI), cardiovascular risk factors, comorbid conditions, New York Heart Association (NYHA) functional class, EuroSCORE II, and preoperative echocardiographic data. Twelve-lead electrocardiography and chest radiography were also performed. A preoperative coronary angiography and/or coronary computed tomography angiography (CCTA) were performed before the operation.

In patients with ischemic cardiomyopathy, a myocardial viability test (Cardiac Magnetic Resonance, CMR) was performed if significant extensive areas of myocardial akinesia were found at echocardiogram. Early postoperative data were collected and significant perioperative complications were described. A 5.2 (3.5–7.5) years complete clinical and echocardiographic follow-up was performed. At follow-up, mortality, cardiovascular mortality, incidence of cardiovascular events (myocardial infarction, stroke), rehospitalization for heart failure, NYHA functional class, and recurrence of MR were evaluated. Moreover, LVEF and LV volumes were also compared.

### Statistical Analysis

Categorical variables are expressed as frequencies (percentage), and continuous variables are described as median values with the interquartile range (25th–75th). Differences in categorical variables were calculated using the Chi-square test or Fisher’s exact test; the Wilcoxon test was used for continuous variables. Survival analysis was performed by Kaplan–Meier curves.

Statistical significance was set at *p* < 0.05. All analyses were performed using SAS software (version 9.4, SAS Institute, Cary, NC, USA) and MedCalc Statistical Software, version 20.113, MedCalc Software Ltd., Ostend, Belgium; “https://www.medcalc.org”.

## 3. Results

In the study period, 96 patients underwent MV repair or replacement for disproportionate (N = 61, 64%) or proportionate (N = 35, 36%) secondary MR.

Patients characteristics are summarized in Table 1. Age was 72 (63, 76) years, with 62% males, for the disproportionate group vs. 64 (60, 72) years, 97% males, in the proportionate group, *p* = 0.05. Cardiovascular risk factors (hypertension, diabetes, dyslipidemia, and history of a smoking habit) were comparable between the two groups. Ischemic dilated cardiomyopathy was diagnosed in 34 (55%) patients of the disproportionate group and in 16 (45%) patients of the proportionate group (*p* = 0.4). Non-ischemic dilated cardiomyopathy was observed in 27 (45%) patients and 19 (55%) patients, respectively (*p* = 0.4). CMR was performed in six patients (three for each group). Neither extensive areas of transmural late gadolinium enhancement nor significant thinning (<5 mm) of the anterior myocardial wall in the end-diastolic phase were found.

Twenty-nine (47%) patients in the disproportionate group vs. 16 (46%) patients in the proportionate group were in NYHA functional class ≥ III, *p* = 0.6. Most importantly, as expected, patients of the disproportionate group presented a higher degree of MR based on EROA compared to the proportionate group (0.35 vs. 0.22 cm^2^, respectively, *p*= <0.001) with less LV dilatation (LVEDV 129 vs. 214 mL, respectively, *p*= < 0.0001).

Operative risk (EuroSCORE II) was comparable between the two groups, even if higher in the proportionate group. In the disproportionate MR group, 16 (27%) patients underwent MV replacement and 45 (74%) patients underwent MV repair. In the proportionate MR group, 7 (21%) patients underwent MV replacement and 28 (79%) patients underwent MV repair. In the disproportionate group, 27 (44%) patients underwent combined procedures (other than aortic valve surgery); of these, 13 (21%) underwent CABG. In the proportionate group, 11 (31%) were combined procedures; of these, 5 (14%) underwent CABG. Twenty-seven (44%) patients in the disproportionate group and 10 (28%) patients in the proportionate group received a tricuspid valve repair by either prosthetic annular reshaping or the De Vega technique [21].

The CPB time was 135 (115, 163) minutes in the disproportionate group vs. 166 (143, 197) minutes in the proportionate group (*p* = 0.01). The aortic cross-clamping time was 101 (80, 122) minutes vs. 133 (111, 155) minutes, respectively (*p* = 0.001) (Table 2).

Thirty-day mortality was comparable in the two groups; indeed, the overall survival was 100%. Mediastinal revision for bleeding was required in three (5%) patients of the disproportionate group vs. two (6%) patients of the proportionate group. Postoperative atrial fibrillation was observed in eight (13%) vs. six (17%) patients, respectively. Two patients of the proportionate group required pacemaker implantation, and in one patient a postoperative stroke occurred early after the operation.

**Figure 1:**
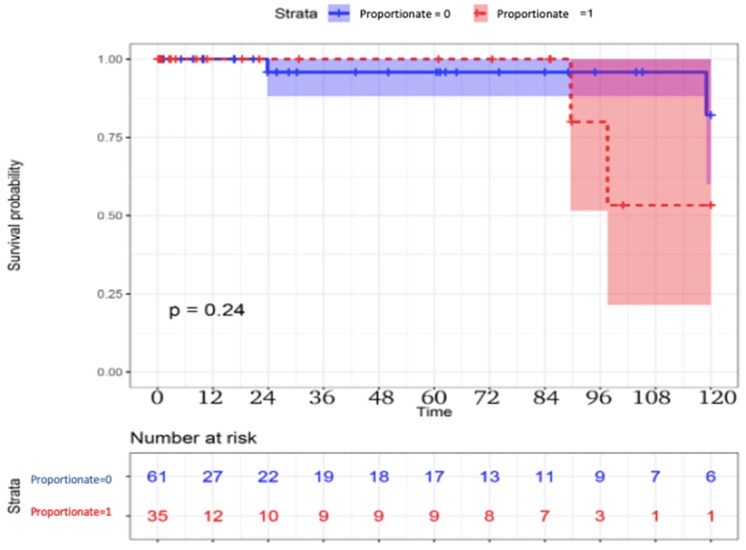
Survival curves between proportionate vs. disproportionate groups.

The long-term follow-up results are reported in Table 3. Late mortality was higher in the proportionate group. Three (5%) patients died in the disproportionate group vs. four (11%) patients in the proportionate group (*p* = 0.2), with a possible improved survival in the disproportionate group, although not reaching statistical significance (Figure 1). Cardiovascular mortality also differed between the disproportionate and proportionate MR groups, two (3%) patients vs. three (9%) patients, respectively, *p* = 0.5. In the disproportionate group, one patient died of oncological disease, one patient died of sudden cardiac death with cardiac arrest, and one patient died of progressive heart failure. In the proportionate group, one patient died of oncological disease, and three patients died of progressive heart failure. New cardiovascular events occurred in two (3%) vs. two (6%) patients, respectively. *p* = 0.5.

A significant difference was observed in terms of rehospitalization for heart failure and NYHA functional class. Rehospitalization for heart failure was observed in four (6%) patients in the disproportionate group, vs. seven (20%) patients of the proportionate group, *p* = 0.04. An NYHA functional class ≥ III was observed in five (8%) patients vs. nine (26%) patients, respectively, *p* = 0.01. LV volumes were lower in the disproportionate group, with a higher LVEF compared to the proportionate group. The LVEDV was 115 (81, 125) ml vs. 168 (128, 188) ml, respectively, *p* < 0.001. The LVESV was 48 (34, 69) ml vs. 97 (60, 122) ml, respectively, *p* < 0.001. The LVEF was 52 (42, 60)% vs. 39 (34, 48)%, respectively, *p*= 0.03. No cases of recurrent MR of a more than moderate grade were observed in the two groups.

## 4. Discussion

Chronic MR is the second most common valve disease in Europe [22]. MR can be defined as primary or secondary (functional). In primary or organic MR, valve insufficiency is caused by structural abnormalities of the valve leaflets and subvalvular apparatus. In secondary MR, the MV anatomy is normal with no sign of organic disease, and the MR is related to mechanical or electrical abnormalities of the LV and/or the left atrium. In these patients, in case of LV dysfunction, MR is a consequence of increasing tethering forces caused by lateral and apical papillary muscle displacement, leading to reduced closing forces, annulus dilatation and flattening, and the loss of leaflet coaptation [22,23,24]. In relation to the different pathophysiological mechanism of MR of atrial origin, as in the case of chronic atrial fibrillation, this study focused on MR of ventricular origin. Patients presented signs and symptoms of heart failure and dilated cardiomyopathy with systolic dysfunction. The dilated cardiomyopathy could be of either ischemic (early after acute myocardial infarction and/or chronic coronary syndrome) or non-ischemic origin [25,26].

The benefit of the surgical treatment of primary MR is well established [27]. Conversely, the surgical treatment of secondary MR, largely related to LV dilatation and systolic dysfunction, is still controversial regarding the possible benefits of MV surgery and in many cases unsatisfactory. Indeed, only 5–22% of these patients undergo MV repair or replacement [28,29]. Furthermore, the promising recent results of transcatheter mitral valve edge-to-edge repair (M-TEER) meet a growing interest in the percutaneous treatment of these patients [30,31,32]. In the era of personalized medicine, patient selection aimed at identifying the subset of patients with functional MR who may benefit most from MV treatment seems to represent useful information for preoperative risk stratification [1,2,3,4,5,6,7,8,9,33,34,35,36,37,38].

The two previous randomized studies about indications for the percutaneous treatment of functional MR (patients not referred to cardiac surgery) were the MITRA-FR trial (Percutaneous Repair with the Mitra- Clip Device for Severe Functional/Secondary Mitral Regurgitation) and the COAPT trial (Cardiovascular Outcomes Assessment of the MitraClip Percutaneous Therapy for Heart Failure Patients with Functional Mitral Regurgitation) [11,12].

In these trials, the authors randomly assigned patients with chronic heart failure, a reduced LVEF, and severe secondary MR to transcatheter MV repair or to a control group that did not receive the procedure.

In the MITRA-FR trial after 12 months, the results of patients who were assigned to transcatheter MV repair were similar to those in the control group with respect to the risk of death or the risk of hospitalization for heart failure. Conversely, in the COAPT trial after two years, patients who were assigned to the intervention group, showed a lower risk of death from any cause and a lower risk of hospitalization for heart failure.

To explain these different results in two similar trials, it is essential to consider that the studies enrolled two distinctly different type of patients. The MITRA-FR investigators enrolled patients who had a striking LV dilatation but relatively modest degrees of MR (EROA 31 mm^2^, RVol 45 mL, LVEDV index 135 mL/m^2^). In contrast, the COAPT investigators primarily enrolled patients in whom the degree of MR was disproportionately great compared to the lower degree of LV enlargement (EROA 41 mm^2^, RVol 60 mL, LVEDV index 101 mL) [11,12,13].

The seemingly contradictory outcomes of the two recent randomized controlled trials were explained by the innovative conceptual framework proposed by Grayburn et al. [13]. Patients who had a MR proportional to the degree of LV dilatation met the inclusion criteria of the MITRA-FR trial. During long-term follow-up, the LVEDV and clinical outcomes did not differ from those of medically treated control subjects. In contrast, the COAPT trial participants had 30% smaller LV volumes but an EROA that was 30% higher compared to those of the MITRA-FR trial, indicating that they were affected by a disproportionate MR. In these patients, transcatheter MV repair decreased the risk of death and hospitalization for heart failure, and a significant decrease in LVEDV accompanied these benefits. In the present study, we first applied this framework to patients who underwent surgical MV repair or replacement. As a result, it appears that determining whether the severity of MR is proportionate to the LVEDV seems relevant to select the best treatment for patients with chronic heart failure and systolic dysfunction [13].

In relation to the different inclusion and exclusion criteria of COAPT and MITRA-FR, and regardless of the reasons for choosing these criteria, the clinical characteristics of the patients included in the two trials were different, and this is an indicator of the importance in patient selection to identify the patient who can benefit most from MV treatment.

A direct comparison between patients with disproportionate vs. proportionate MR has rarely been performed, and so far the data are still scarce. Only limited data and results from a few previous studies are available, mainly in the setting of transcatheter procedures. Despite the controversial results, an advantage in patients with disproportionate MR seems conceivable [39,40]. Indeed, in disproportionate MR the severity of valve dysfunction is excessive relative to the LV dilation, suggesting that the valve lesion contributes independently and significantly to the hemodynamic burden. In these patients, MR is not simply a marker of LV dysfunction but a key contributor to volume overload, elevated filling pressures, and progressive remodeling. In these patients, MV intervention can offload the ventricle, reduce symptoms, improve forward output, and potentially modify the heart failure’s progression. This concept was underscored by the divergent outcomes of the COAPT and MITRA-FR trials, highlighting the importance of patient selection and the relative contribution of MR to the overall disease state. However more evidence is needed to outline a possible real superiority of disproportionate patients in terms of late outcome.

The role of surgery in secondary MR was considered in the latest Guidelines [19]. According to the 2021 ESC/EACTS Guidelines on the Management of Valvular Heart Disease, surgery is not routinely recommended for all patients. Instead, MV surgery is recommended in patients with severe secondary MR undergoing CABG or other cardiac surgery. Isolated MV surgery may be considered in symptomatic patients with persistent severe MR despite optimal medical therapy and CRT (when indicated), after the Heart Team evaluation. Regarding the surgical strategy, Goldstein et al. [6], investigated the outcomes of two surgical approaches (MV repair or replacement) in patients with severe ischemic MR. At a two-year follow-up, there were no significant differences in terms of survival between the two groups. However, recurrent MR was significantly more frequent after repair, leading to more heart failure events compared to MV replacement. These results challenged the prior preference for repair in this setting and suggested that MV replacement may offer more durable results in selected patients. In the case of valve replacement, the need to preserve the valve leaflets and the subvalvular apparatus is considered essential to preserve the geometry and structure of the LV [6,41,42,43,44]. The indication for surgery in these patients should remain Guideline-oriented and Heart Team-oriented. However, also applying the Grayburn framework to cardiac surgery patients [13] could help one to realize if one of the two categories (disproportionate or proportionate MR) really presents a superior advantage in the surgical correction of MR. In this exploratory study, a possible survival benefit, even if not statistically significant, was observed in patients with disproportionate MR. Nevertheless, a significant difference in terms of rehospitalization for heart failure, NYHA functional class, and LVEF and LV volumes was observed in the disproportionate group, with a better prognosis after the surgical correction of MR. It would seem that patients with disproportionate MR respond more favorably to MV surgery; however larger and multicenter studies are required to confirm these observational findings.

## 5. Conclusions

Patients with LV dilatation and dysfunction seem to respond favorably to MV surgery if they exhibit degrees of MR that are disproportionately greater than might be expected from the degree of LV enlargement. In the field of functional MR, in cases where cardiac surgery is indicated and in which MR should be corrected, the identification of those patients who may benefit most from surgical treatment seems useful to recognize high-risk patients and to determine if an intervention to the MV would significantly improve their clinical outcome and prognosis. Further studies with larger series of data are required to provide definitive results, with the possible stratification of further subgroups of patients.

## 6. Limitations

This preliminary study was conceived to apply this framework to surgical patients. This study has some limitations, such as the ambispective nature of the study, with a limited number of patients, who inevitably present a certain degree of clinical and procedural heterogeneity. However, selecting retrospectively an adequate number of patients with pure functional MR, without the slightest sign of MV organic disease or other etiologies, significantly limited the number of patients eligible for enrollment. Furthermore, in this study a possible difference between MV replacement or MV repair was not investigated, both because the limited number of patients does not allow us to stratify by type of operation, and because the study was focused on the long-term clinical outcome of patients undergoing the surgical correction of MR, regardless of the type of technique.

## Figures and Tables

**Table 1:** Patients’ characteristics.

	Disproportionate N = 61	Proportionate N = 35	*p*-Value
Age, median (IQR) years	72 (63, 76)	64 (60, 72)	0.05
Male gender, n (%)	38 (62)	34 (97)	<0.001
BMI, median (IQR) Kg/m^2^	70 (52, 81)	76 (60, 84)	0.05
Hypertension, n (%)	47 (77)	26 (74)	0.8
Dyslipidemia, n (%)	33 (54)	18 (51)	0.9
Diabetes, n (%)	20 (33)	11 (31)	0.9
History of smoking habit, n (%)	23 (38)	17 (49)	0.6
Renal failure, n (%)	8 (8)	4 (11)	0.8
History of AF (other than chronic), n (%)	33 (54)	12 (34)	0.06
PVD, n (%)	5 (8)	4 (11)	0.8
Neurovascular events, n (%)	4 (6)	3 (8)	0.9
COPD, n (%)	6 (10)	4 (11)	0.6
EuroSCORE II, median (IQR)%	2 (1, 3)	3 (1, 4)	0.5
MI in previous three months, n (%)	18 (30)	5 (14)	0.09
Non-ischemic dilated CM, n (%)	27 (45)	19 (55)	0.4
Ischemic dilated CM, n (%)	34 (55)	16 (45)	0.4
NYHA ≥ III, n (%)	29 (47)	16 (46)	0.6
CRT, n (%)	11 (18)	4 (22)	0.6
Mitral annulus, median (IQR) mm	39 (34, 42)	39 (38, 41)	0.8
LVEF, median (IQR)%	48 (42, 55)	39 (32, 50)	0.003
PASP, median (IQR)%	40 (31, 50)	40 (30, 45)	0.9
TAPSE, median (IQR) mm	20.0 (17, 25)	22 (18, 25)	0.9
Moderate or severe TR, n (%)	27 (44)	10 (29)	0.1
RVol, median (IQR) ml	63 (40, 72)	42 (32, 56)	<0.001
EROA, median (IQR) cm^2^	0.35 (0.3, 0.4)	0.22 (0.2, 0.3)	<0.001
LVEDV, median (IQR) mL	129 (102, 155)	214 (168, 232)	<0.001
LVESV, median (IQR) mL	68 (47, 86)	121 (89, 144)	<0.001
EROA/LVEDV (mean ± SD) mm^2^/mL	0.26 ± 0.06	0.10 ± 0.02	0.01

BMI: body mass index; MI: myocardial infarction; AF: atrial fibrillation; CM: cardiomyopathy; CRT: cardiac resynchronization therapy; PVD: peripheral vascular disease; COPD: chronic obstructive pulmonary disease; LVEF: left ventricular ejection fraction; PASP: pulmonary artery systolic pressure; TAPSE: tricuspid annular plane systolic excursion; TR: tricuspid regurgitation; EROA: effective regurgitant orifice area; LVEDV: left ventricular end diastolic volume; LVESV: left ventricular end systolic volume. SD: standard deviation.

**Table 2:** Operative data.

	Disproportionate (N = 61)	Proportionate (N = 35)	*p* Value
MV repair, n (%)	45 (74)	28 (79)	0.5
MV replacement, n (%)	16 (27)	7 (21)	0.5
Bioprosthesis n (%)	12(20)	5 (14)	0.5
Mechanical prosthesis, n (%)	4 (6)	2 (6)	0.8
Combined procedures, n (%)	27 (44)	11 (31)	0.2
Concomitant CABG, n (%)	13 (21)	5 (14)	0.5
Concomitant TV repair, n (%)	27 (44)	10 (28)	0.1
Prosthetic ring, n (%)	7 (11)	1 (3)	0.2
De-Vega, n (%)	20 (33)	9 (26)	0.6
CPB time, median (IQR) minutes	135 (115, 163)	166 (143, 197)	0.01
ACC time, median (IQR) minutes	101 (80, 122)	133 (111, 155)	0.001

MV: mitral valve; CABG: coronary artery bypass grafting; TV: tricuspid; CPB time: cardiopulmonary bypass time; ACC: aortic cross-clamping time.

**Table 3:** Long-term outcome.

	Disproportionate N = 61	Proportionate N = 35	*p* Value
30-day mortality, n (%)	0 (0)	0 (0)	-
Follow-up, median (IQR) years	5.1 (3.4–7.5)	5.3 (3.7–7.8)	0.6
5-year FU mortality, n (%) MV repair, n (%) MV replacement, n (%)	3 (5) 2 (3) 1 (2)	4 (11) 3 (9) 1 (3)	0.2
Cardiovascular mortality, n (%) MV repair, n (%) MV replacement, n (%)	2 (3) 1 (2) 1 (2)	3 (9) 2 (6) 1 (3)	0.5
Cardiovascular events, n (%) MV repair, n (%) MV replacement, n (%)	2 (3) 0 (0) 2 (3)	2 (6) 1 (3) 1 (3)	0.5
Rehospitalization for heart failure, n (%) MV repair, n (%) MV replacement, n (%)	4 (6) 1 (2) 3 (5)	7 (20) 5 (14) 2 (6)	0.04
NYHA ≥ III, n (%) MV repair, n (%) MV replacement, n (%)	5 (8) 2 (3) 3 (5)	9 (26) 5 (14) 4 (11)	0.01
LVEF, median (IQR) %	52 (42, 60)	39 (34, 48)	0.03
LVEDV, median (IQR) ml	115 (81, 125)	168 (128, 188)	<0.001
LVESV, median (IQR) ml	48 (34, 69)	97 (60, 122)	<0.001
MR recurrence (more than moderate) n (%)	0 (0)	0 (0)	-

FU: follow-up; NYHA: New York Heart Association functional class; LVEF: left ventricular end-diastolic volume.

## Data Availability

The original contributions presented in this study are included in the article. Further inquiries can be directed to the corresponding author.

## Abbreviations

The following abbreviations are used in this manuscript:

AF	Atrial fibrillation
CABG	Coronary artery bypass grafting
COPD	Chronic obstructive pulmonary disease
CM	Cardiomyopathy
CMR	Cardiac Magnetic Resonance
CPB	Cardiopulmonary bypass
CV	Cardiovascular
EROA	Effective regurgitant orifice area
GDMT	Guideline-directed medical therapy
LV	Left ventricular
LVEDD	Left ventricular end-diastolic diameter
LVESD	Left ventricular end-systolic diameter
LVEDV	Left ventricular end-diastolic diameter
LVESD	Left ventricular end-systolic diameter
LVEF	Left ventricular ejection fraction
MR	Mitral regurgitation
MV	Mitral valve
NYHA	New York Heart Association
PVD	Peripheral vascular disease
RVol	Regurgitant volume
